# Ethnic differences in body fat distribution among Asian pre-pubertal children: A cross-sectional multicenter study

**DOI:** 10.1186/1471-2458-11-500

**Published:** 2011-06-26

**Authors:** Ailing Liu, Nuala M Byrne, Masaharu Kagawa, Guansheng Ma, Kallaya Kijboonchoo, Lara Nasreddine, Bee Koon Poh, Mohammad Noor Ismail, Andrew P Hills

**Affiliations:** 1 National Institute for Nutrition and Food Safety, Chinese Center for Disease Control and Prevention, Beijing, China; 2 Griffith Health Institute, Griffith University and Mater Mother's Hospital, Mater Medical Research Institute, Australia; 3 Institute of Nutrition Sciences, Kagawa Nutrition University, Saitama, Japan; 4 Institute of Nutrition, Mahidol University at Salaya, Nakhon Pathom, Thailand; 5 Department of Nutrition and Food Sciences, Faculty of Agriculture and Food Sciences, American University of Beirut, Beirut, Lebanon; 6 Department of Nutrition and Dietetics, Faculty of Health Sciences, Universiti Kebangsaan Malaysia, Kuala Lumpur, Malaysia

**Keywords:** body fat distribution, ethnicity, children

## Abstract

**Background:**

Ethnic differences in body fat distribution contribute to ethnic differences in cardiovascular morbidities and diabetes. However few data are available on differences in fat distribution in Asian children from various backgrounds. Therefore, the current study aimed to explore ethnic differences in body fat distribution among Asian children from four countries.

**Methods:**

A total of 758 children aged 8-10 y from China, Lebanon, Malaysia and Thailand were recruited using a non-random purposive sampling approach to enrol children encompassing a wide BMI range. Height, weight, waist circumference (WC), fat mass (FM, derived from total body water [TBW] estimation using the deuterium dilution technique) and skinfold thickness (SFT) at biceps, triceps, subscapular, supraspinale and medial calf were collected.

**Results:**

After controlling for height and weight, Chinese and Thai children had a significantly higher WC than their Lebanese and Malay counterparts. Chinese and Thais tended to have higher trunk fat deposits than Lebanese and Malays reflected in trunk SFT, trunk/upper extremity ratio or supraspinale/upper extremity ratio after adjustment for age and total body fat. The subscapular/supraspinale skinfold ratio was lower in Chinese and Thais compared with Lebanese and Malays after correcting for trunk SFT.

**Conclusions:**

Asian pre-pubertal children from different origins vary in body fat distribution. These results indicate the importance of population-specific WC cut-off points or other fat distribution indices to identify the population at risk of obesity-related health problems.

## Background

Given the high and rapidly increasing prevalence of pediatric obesity and obesity-related health risks such as hypertension, metabolic syndrome, and type 2 diabetes in both developed and developing countries [1-4], body fat distribution, in addition to total body fat, is an important issue. Body mass index (BMI) and percentage of body fat (%BF) do not fully explain the ethnic differences in cardiovascular morbidity and diabetes [5], despite the latter being well documented [6-9]. An android fat pattern and central fat deposition is a stronger predictor of cardiovascular disease, type 2 diabetes and metabolic risk factors than overall adiposity in both adults and children [10-13].

Some previous studies investigated the ethnic difference in fat distribution in terms of a trunk and extremity model [11,13,14], upper and lower body fat model [15-17], and subcutaneous and visceral abdominal adipose tissue model [14,18-20], among Caucasians, blacks and Asian adults and children. Asians and blacks appear to have more central fat, upper body fat, subcutaneous and visceral abdominal adipose tissue than Caucasians. However the ethnic difference in fat distribution is less well understood, especially in children and adolescents due to the effect of sexual maturation on body composition [21]. Moreover, some studies also report different fat patterns among Asian adults from different origins [22]. However, to our knowledge, few data are available on the difference in fat distribution across Asian children from different backgrounds. A lack of understanding of ethnic differences in body fat distribution may result in the misuse or misinterpretation of results obtained from anthropometric indices. Data on body fat distribution of individuals from different ethnicities is necessary to better understand the risk of cardiovascular risk factor clustering in children. Accordingly, the purpose of the current study was to investigate the influence of ethnicity on body fat patterns in Asian pre-pubertal children from four distinctly different origins.

## Methods

### Participants

Participants from four Asian countries were involved including one East Asian country (China), one West Asian country (Lebanon), and two South-East Asian countries (Malaysia and Thailand). A non-random purposive sampling approach was used to recruit participants. In each country, 5-8 schools were selected and an advertisement sent to all 8-10 year-old students and their parents. Children who met the inclusion criteria (Tanner stage 1 of puberty and free from any diagnosed medical condition that might potentially interfere with body composition measurement) were enrolled in the study. In addition, given that fat distribution may differ with BMI status, a number of overweight and obese children (defined by WHO BMI classification for school-aged children and adolescents) were purposively recruited in each country. A total of 922 participants were recruited and 758 participants (220 Chinese, 154 Lebanese, 197 Malay and 187 Thai) completed all assessments. Ethnicity was determined by self-identification and by parents of participants who identified as having the same origin.

Ethical clearance was obtained from the relevant committee in each participating country: Ethical Review Committee of National Institute for Nutrition and Food Safety, Chinese Center for Disease Control and Prevention, China; Institutiona Review Board, American University of Beirut, Lebanon; Medical Research and Ethics Committee of the Universiti Kebangsaan Malaysia, Malaysia; and Mahidol University Institution Review Board, Thailand. The study protocol was explained to the parent(s) and the children and written consent obtained from each child and/or their parent(s).

### Anthropometric measurements

Height was measured to the nearest 0.1 cm using a portable stadiometer (Holtain, Crymych, UK). Fasting body weight was measured using a SECA^TM ^electronic scale (Hamburg, Germany) to the nearest 0.1 kg with participants wearing only underwear after urinating in the early morning. BMI (kg/m^2^) was then calculated as weight (kg) divided by the square of height (m). Waist circumference (WC) was measured using a tape to the nearest 0.1 cm midpoint between the lower costal border and the top of the iliac crest, and the measurement was taken at the end of normal expiration. Pubertal stage (genitalia development and pubic hair in boys, breast development and pubic hair in girls) was assessed according to the criteria of Tanner [23] by trained investigators in each country.

### Skinfold measurements

Skinfold thickness (SFT) was measured using a Holtain T/W skinfold caliper (Holtain Ltd., Crosswell, Crymych, Pembs, SA41 3UF, UK) to the nearest of 0.1 mm according to the International Society for the Advancement of Kinanthropometry (ISAK) protocol [24]. Measurement sites included five skinfolds: biceps (the most anterior surface of the arm in the mid-line at the level of the mid-acromiale-radiale landmark), triceps (the most posterior surface of the arm over the triceps muscle), subscapular (2 cm along a line running laterally and obliquely downward from the subscapular landmark at a 45° angle), supraspinale (the line from the marked iliospinale to the anterior axillary border which intersects with the horizontal line at the superior border of the ilium), and medial calf (the most medial aspect of the calf at the level where it has maximal circumference). All measurements were taken twice and the mean was calculated. If the difference between the two measurements was more than 0.5 mm a third measurement was taken and the median was regarded as the skinfold thickness.

Trunk SFT (sum of subscapular and supraspinale) and upper extremity (sum of biceps and triceps) was used as the index for the absolute amount of trunk fat and upper extremity fat, respectively. WC was used as the indicator of the absolute amount of abdominal fat. Trunk/upper extremity ratio and supraspinale/upper extremity ratio were used as the indices for the relative distribution of fat mass in the trunk and extremities. Higher trunk SFT, WC, trunk/upper extremity ratio or supraspinale/upper extremity ratio indicates more trunk fat or central depots. Supraspinale/subscapular ratio was used to describe the upper to lower trunk fat distribution pattern.

### Body fat assessment

The isotope deuterium dilution technique was used to predict FM from TBW and fat-free mass (FFM) values. In brief, a 5 mL sample of urine was collected before consuming a dose of the isotope to determine the basal deuterium level in the body. A 10% deuterium oxide dose of 0.5 g per kg body weight was given orally. A second urine sample was collected 5 h later to allow complete equilibration within the body water compartments. All urine samples from the four countries were sent to the laboratory at Queensland University of Technology, Australia for analysis. The enrichment of the pre-dose urine sample, post-dose urine sample, the dose given and the local tap water were measured by isotope ratio mass spectrometry (20:20 Hydra Model, PDZ Europa, Crewe, UK) and TBW determined. FFM was then derived from TBW using the Lohman's age- and gender-specific constants for hydration of FFM for children [25]. The absolute FM was derived by subtracting FFM from weight, based on the two-compartment body composition model.

In order to harmonize the data collection among the four countries, two training workshops were held for research staff from each centre. Moreover, the same equipment and procedures were employed in each country with all measurements taken following standardized operating procedures by the trained investigators.

### Statistical analysis

All variables were transformed to achieve the normal distribution before analysis wherever necessary using natural logarithms. One-way analysis of variance was used to test difference in base characteristics and anthropometric parameters between sex and among ethnic groups. General linear model of analysis of covariance (ANCOVA) was employed to test the ethnic differences in body fat variables with Bonferroni multiple comparisons. The comparison of WC among ethnic groups was tested by ANCOVA adjusted for BMI and age. The comparison of SFT and trunk/extremity ratios among different groups was tested by ANCOVA adjusted for total body fat. The comparisons of upper/lower trunk fat ratio among different groups were tested by ANCOVA adjusted for trunk SFT. All statistical analyses were performed using SPSS version 13.0 (SPSS, Inc., Chicago, IL, USA). A two-tailed *P *< 0.05 was considered significant.

## Results

Descriptive characteristics of the participants are presented in Table 1 by sex-ethnic subgroups. All participants were in the BMI range 12.2 to 34.9 kg/m^2^. There was a significant difference in height, weight, BMI and FM among Chinese, Lebanese, Malays and Thais in each sex except for weight, BMI and FM in girls. At a given BMI and age, boys from each country had a similar FM (*P *= 0.371), while there was a marginal significant difference in BMI and age-corrected FM among girls from the four countries (*P *= 0.047).

**Table 1:** Descriptive characteristics of the participants

	Chinese	Lebanese	Malay	Thai	*P *value
Boys					
N	127	74	105	83	
Age (yr)	9.0 ± 0.8	9.0 ± 0.7	9.0 ± 0.8	8.8 ± 0.8	0.327
Height (cm)	138.4 ± 7.7	134.8 ± 8.0	131.4 ± 7.0	133.2 ± 5.9	<0.001
Weight (kg)	38.2 ± 10.3	34.7 ± 9.7	33.1 ± 12.0	30.0 ± 6.0	<0.001
BMI (kg/m^2^)	19.6 ± 3.7	18.8 ± 3.7	18.8 ± 5.3	16.8 ± 2.5	<0.001
FM (kg)	11.4 ± 6.2	9.9 ± 6.3	10.0 ± 7.8	7.4 ± 3.7	<0.001
FM_adjusted _(kg)	10.0 ± 0.2	9.7 ± 0.3	9.7 ± 0.2	10.2 ± 0.2	0.371
Girls					
N	93	80	92	104	
Age (yr)	9.0 ± 0.8	8.8 ± 0.7	9.0 ± 0.8	8.8 ± 0.8	0.285
Height (cm)	136.3 ± 7.9	133.4 ± 7.9	133.0 ± 9.5	133.6 ± 8.2	0.023
Weight (kg)	34.0 ± 9.1	31.0 ± 7.4	33.6 ± 12.0	31.9 ± 8.6	0.099
BMI (kg/m^2^)	18.1 ± 3.4	17.2 ± 2.7	18.6 ± 4.7	17.6 ± 3.3	0.083
FM (kg)	10.0 ± 5.3	8.7 ± 4.3	10.9 ± 7.2	10.0 ± 4.8	0.070
FM_adjusted _(kg)	9.7 ± 0.2	9.7 ± 0.2	9.9 ± 0.2	10.4 ± 0.1	0.047

FM_adjusted: _FM adjusted for age and BMI. Values were described as mean ± standard deviation except for FM_adjusted _(mean ± standard error)

### Skinfold thickness

There was a significant ethnic difference in biceps, triceps, subscapular, supraspinale, and medial calf SFT among Chinese, Lebanese, Malays, and Thais in each sex (*P *< 0.001) after adjustment for age and FM except for subscapular SFT in boys (Table 2).

**Table 2:** Comparison of subcutaneous adiposity and fat distribution variables among four ethnic groups by sex (adjusted mean ± standard error)

	Chinese	Lebanese	Malays	Thais	Bonferroni comparisons
Boys					
Waist circumference (cm)^※^	66.7 ± 0.3^§^	64.1 ± 0.4	62.6 ± 0.3^§^	65.9 ± 0.4	*P *< 0.001 C = T > L > M
Skinfold					
Biceps (mm)^#^	7.9 ± 0.2	9.1 ± 0.3^§^	9.5 ± 0.2	6.6 ± 0.3	*P *< 0.001 C = L = M > T
Triceps (mm)^#^	15.5 ± 0.3	11.9 ± 0.4	13.0 ± 0.3	12.3 ± 0.4^§^	*P *< 0.001 C > L = M = T
Subscapular (mm)^#^	12.1 ± 0.4	11.4 ± 0.5	12.7 ± 0.4	-	*P *= 0.115
Supraspinale (mm)^#^	15.1 ± 0.4	9.4 ± 0.5	12.1 ± 0.4	12.0 ± 0.5	*P *< 0.001, C > M = T > L
Medial calf (mm)^#^	-	14.1 ± 0.4	11.8 ± 0.3	10.1 ± 0.4^§^	*P *< 0.001, L > M >T
Trunk (mm) ^#^	28.0 ± 0.7	21.6 ± 0.9	25.6 ± 0.8	-	*P *< 0.001, C = M > L
Trunk/upper extremity ratio ^#^	1.15 ± 0.02	0.92 ± 0.03	0.99 ± 0.02	-	*P *< 0.001, C > M > L
Supraspinale/upper extremity ratio ^#^	0.62 ± 0.01	0.42 ± 0.02^§^	0.48 ± 0.0^§^	0.60 ± 0.02	*P *< 0.001, C = T > M > L
Subscapular/supraspinale ratio^†^	0.88 ± 0.03	1.17 ± 0.04^§^	1.09 ± 0.04^§^	-	*P *< 0.001, C < L = M
Girls					
Waist circumference (cm)^※^	63.4 ± 0.3	61.9 ± 0.3	59.7 ± 0.3	64.0 ± 0.3	*P *< 0.001, C = T > L > M
Skinfold					
Biceps (mm)^#^	7.7 ± 0.2	8.2 ± 0.2	9.7 ± 0.2	6.7 ± 0.2	*P *< 0.001, M > L > C = T
Triceps (mm)^#^	15.2 ± 0.3	11.7 ± 0.3	13.1 ± 0.3	13.2 ± 0.3	*P *< 0.001, C > M = T > L
Subscapular (mm)^#^	11.4 ± 0.3	9.8 ± 0.3	11.3 ± 0.3	-	*P *= 0.003, C = M > L
Supraspinale (mm)^#^	14.9 ± 0.4	10.0 ± 0.4	12.6 ± 0.4	13.1 ± 0.4	P <0.001, C > M = T > L
Medial calf (mm)^#^	-	14.5 ± 0.4	13.5 ± 0.3	11.9 ± 0.3	*P *= 0.009, L > M = T
Trunk (mm) ^#^	26.2 ± 0.6	19.7 ± 0.7	23.9 ± 0.6	-	*P *< 0.001,C > M > L
Trunk/upper extremity ratio ^#^	1.13 ± 0.02	0.94 ± 0.02	0.99 ± 0.02	-	*P *< 0.001,C > L = M
Supraspinale/upper extremity ratio ^#^	0.63 ± 0.01	0.47 ± 0.01	0.52 ± 0.01	0.64 ± 0.01	*P *< 0.001, C = T > M > L
Subscapular/supraspinale ratio^†^	0.84 ± 0.02	1.06 ± 0.03	0.98 ± 0.03	-	*P *< 0.001, L = M > C

^§ ^Significant difference in adjusted mean value between boys and girls within each ethnic group using ANCOVA with Bonferroni multiple comparisons procedure

^※^Adjusted for BMI and age

^# ^Adjusted for body fat mass and age

^† ^Adjusted for trunk fat

C: Chinese; L: Lebanese; M: Malays; T: Thais

### Central fat distribution pattern

There was a significant ethnic difference in trunk SFT among Chinese, Lebanese and Malays in each sex after adjustment for age and FM (*P *< 0.001) (Table 2). In boys, Chinese and Malays had a similar trunk SFT and both had a higher value than the Lebanese. Chinese girls had a higher trunk SFT than Malays, who in turn were higher than the Lebanese.

Significant differences in WC among ethnic groups was found in both boys and girls for a given BMI and age (*P *< 0.001) (Table 2). Chinese boys and girls had a similar WC to their Thai counterparts and both had a higher WC compared with their Lebanese counterparts, who in turn were higher than Malays for a given BMI and age. There was also a significant ethnic difference in trunk/upper extremity ratio among Chinese, Lebanese and Malays in each sex after adjustment for age and FM (*P *< 0.001) (Table 2). In boys, Chinese had a higher trunk/upper extremity ratio than Malays, who in turn was higher than the Lebanese. In girls, the Chinese had a higher ratio than Lebanese and Malays with no significant differences between the latter two groups.

A significant ethnic difference in supraspinale/upper extremity ratio was also found among Chinese, Lebanese, Malays and Thais in each sex after adjustment for age and FM (*P *< 0.001) (Table 2). Chinese and Thai boys and girls had a similar supraspinale/upper extremity ratio and both had a higher value than Malays and Lebanese.

### Upper/lower trunk fat

Significant differences in subscapular/supraspinale ratio were found among Chinese, Lebanese and Malays after adjustment for the trunk SFT and age (*P *< 0.001) (Table 2). Both Chinese boys and girls had a lower trunk fat-adjusted subscapular/supraspinale ratio compared with their Lebanese and Malays countrerparts.

## Discussion

The current study explored ethnic differences in body fat distribution among the four ethnic groups in terms of absolute skinfold thickness at different sites, WC and the trunk to extremity ratios. Chinese boys and girls had a similar fat distribution pattern with Thai boys and girls and both groups had more trunk fat or central fat depots than Malays and Lebanese. Malays tended to have higher trunk/extremity ratios than Lebanese whereas lower WC than Lebanese at a given BMI.

Fat distribution patterns are more closely associated with cardiovascular disease, type 2 diabetes and metabolic risk factors than overall adiposity in both adults and children and contribute to explanations of ethnic differences in cardiovascular morbidities and diabetes [10-13]. A number of previous studies have compared the body fat distribution patterns in Asians with those in Caucasians and blacks. In general, Asians have more central fat depots and visceral fat accumulation than whites in both children [13,17] and adults [22,26-29] regardless of body composition measurement method used. Asian girls enter puberty earlier than white girls which results in shorter legs due to endplate closure in the long bones. However, trunk length continues to grow indicating a long period of pubescent growth. This apparent longer period of pubescence might contribute to the greater trunk FM in Asian girls [30]. It is difficult to make comparisons regarding differences in fat patterns between Asian and other ethnic groups because of the limited data among Asian children and adolescents from different backgrounds. Several studies in adults have reported on differences among Asians from different origins. For example, Lear et al. [22] reported that Chinese (China, Hong Kong, and Taiwan) had less dual-energy X-ray absorptiometry-derived FM while South Asians (Bangladesh, India, Nepal, Pakistan, and Sri Lanka) had more FM than their European counterparts at the same BMI. South Asians had higher FM and total abdominal adipose tissue (as determined by computed tomography) than Chinese at the same BMI. In another study conducted in Indian, Malay and Chinese Singaporeans, Indians had more central fat than Malays and Chinese, and both Malays and Indians had higher abdominal diameter (as measured at the level of the iliac crests with a ruler and tape) than Chinese [31]. Although different adiposity indices were used, our study confirms the differences in fat distribution among Asian children from different origins.

WC, a simple and practical index of visceral adipose tissue [32,33] has been proposed as effective to predict metabolic risk factors in both children and adults [34-37]. WC is also recognized as a key component of the metabolic syndrome in both children and adults [38,39]. Therefore, some countries have developed their own WC percentiles for children and adolescents. Previous studies developed WC percentiles for Chinese and Malaysian children [40-43]. Compared with white, black and Mexican children, Asian children tended to have a lower WC across ages in each sex. The lower WC in Asians can be explained, in part, by the smaller frame size compared to other ethnic groups. Ehtisham et al. [11] indicated that after controlling for height, weight or BMI, both Asian boys and girls aged 14-17 yr appear to have higher WC than their white counterparts. In contrast, Novotny et al. [15] found that Asian girls (11.8 ± 0.05 y) had lower WC than white and Hispanic children even after controlling for height and weight. The inconsistency in results may be, in part, due to the difference in age or stage of maturation and WC measurement site used (narrowest part of the waist, midway between the 10th rib and the iliac crest for the former study, at the level of umbilicus for the latter study). Ethnic differences in body composition vary according to age or pubertal status [44] and significant differences in WC values measured at different sites have been reported [45]. In addition, the difference in the Asian population in the two studies might be a major contributing factor to the inconsistent findings. The Asians in the former study were South Asians including Indian, Pakistani, Bangladeshi, and Sri Lankan, while those in the latter study were more representative of a general Asian population. The difference in other body composition variables among Asians from different backgrounds has been reported [22] so it may be hypothesized that WC will differ among Asians from different origins. Our study confirms this hypothesis indicating that Chinese and Thai children had higher WC values than the Lebanese at a given BMI and age, who in turn were higher than Malays.

One of the main limitations of the present study was the field measures of body fat distribution used rather than more advanced and specific measures such as dual-energy X-ray absorptiometry, magnetic resonance imaging or computed tomography which may provide additional insight into the racial influence on fat distribution. However, compared to these more expensive, advanced techniques which also require highly trained technicians, the anthropometric measures used are relatively simple and inexpensive. These advantages are not insignificant for epidemiological research and clinical practice as they provide an excellent opportunity to screen people with high risk. Moreover, measurement of subcutaneous fat with skinfold calipers has been shown to correlate well with that measured with computed tomography or magnetic resonance imaging [46,47] and WC correlates well with fat distribution measured by dual-energy X-ray absorptiometry [32]. Another limitation to our study was the non-random population sample in each ethnic group. However, our study sample was purposively recruited to encompass a number of overweight and obese children. Given that fat distribution may differ with BMI status, data based on this sampling method are more convincing.

## Conclusions

The current study indicates that Asian pre-pubertal children from different origins vary in body fat distribution with greater trunk fat depots in Chinese and Thai children compared with Malays who in turn have higher values than Lebanese. These results indicate the importance of population-specific WC cut-off points or other fat distribution indices to identify the population at risk of obesity-related health problems.

## Abbreviations

ANCOVA: Analysis of covariance; BMI: Body mass index; FFM: Fat-free mass; FM: Fat mass; %BF: Percentage of body fat; SFT: Skinfold thickness; TBW: Total body water; WC: Waist circumference

## Competing interests

The authors declare that they have no competing interests.

## Authors' contributions

AL contributed to data collection in China, data analysis and drafted the manuscript. NMB contributed to the design, interpretation of data and revising the manuscript. MK contributed to the analysis of samples and revising the manuscript. GM, KK, LN and MNI were the principal investigators in their respective countries and contributed to data collection and interpretation. BKP contributed to data collection and interpretation in Malaysia and revising the manuscript. APH contributed to the design, interpretation of data and revision of the manuscript. All authors read and approved the final manuscript.

## Pre-publication history

The pre-publication history for this paper can be accessed here:

http://www.biomedcentral.com/1471-2458/11/500/prepub

## References

[B1] Li Y Yang X Zhai F Kok FJ Zhao W Piao J Zhang J Cui Z Ma G Prevalence of the metabolic syndrome in Chinese adolescents Br J Nutr 2008 99 565 570 17662161 10.1017/S0007114507797064

[B2] Li Y Schouten EG Hu X Cui Z Luan D Ma G Obesity prevalence and time trend among youngsters in China, 1982-2002 Asia Pac J Clin Nutr 2008 17 131 137 18364338

[B3] Lobstein T Baur L Uauy R Obesity in children and young people: a crisis in public health Obes Rev 2004 5 Suppl 1 4 85 15096099 10.1111/j.1467-789X.2004.00133.x

[B4] Booth ML Dobbins T Okely AD Denney-Wilson E Hardy LL Trends in the prevalence of overweight and obesity among young Australians, 1985, 1997, and 2004 Obesity 2007 15 1089 1095 10.1038/oby.2007.586 17495184

[B5] McAuley KA Williams SM M JI Goulding A Murphy E Increased risk of type 2 diabetes despite same degree of adiposity in different racial groups Diabetes Care 2002 25 2360 2361 10.2337/diacare.25.12.2360 12453993

[B6] Cossrow N Falkner B Race/ethnic issues in obesity and obesity-related comorbidities J Clin Endocrinol Metab 2004 89 2590 2594 10.1210/jc.2004-0339 15181028

[B7] Ehtisham S Hattersley AT Dunger DB Barrett TG First UK survey of paediatric type 2 diabetes and MODY Arch Dis Child 2004 89 526 529 10.1136/adc.2003.027821 15155395 PMC1719947

[B8] Whincup PH Gilg JA Papacosta O Seymour C Miller GJ Alberti KGMM Cook DG Early evidence of ethnic differences in cardiovascular risk: cross sectional comparison of British South Asian and white children BMJ 2002 324 635 639 10.1136/bmj.324.7338.635 11895820 PMC84394

[B9] Ford ES Prevalence of the metabolic syndrome defined by the International Diabetes Federation among adults in the U.S Diabetes Care 2005 28 2745 2749 10.2337/diacare.28.11.2745 16249550

[B10] Okosun IS Liao Y Rotimi CN Prewitt TE Cooper RS Abdominal adiposity and clustering of multiple metabolic syndrome in White, Black and Hispanic Americans Ann Epidemiol 2000 10 263 270 10.1016/S1047-2797(00)00045-4 10942873

[B11] Ehtisham S Crabtree N Clark P Shaw N Barrett T Ethnic differences in insulin resistance and body composition in United Kingdom adolescents J Clin Endocrinol Metab 2005 90 3963 3969 10.1210/jc.2004-2001 15840754

[B12] Daniels SR Khoury PR Morrison JA The utility of body mass index as a measure of body fatness in children and adolescents: Differences by race and gender Pediatrics 1997 99 804 807 10.1542/peds.99.6.804 9164773

[B13] He Q Horlick M Thornton J Wang J Pierson RN Heshka S Gallagher D Sex and race differences in fat distribution among Asian, African-American, and Caucasian prepubertal children J Clin Endocrinol Metab 2002 87 2164 2170 10.1210/jc.87.5.2164 11994359

[B14] Rush EC Freitas I Plank LD Body size, body composition and fat distribution: comparative analysis of European, Maori, Pacific Island and Asian Indian adults Br J Nutr 2009 102 632 641 10.1017/S0007114508207221 19203416

[B15] Novotny R Going S Teegarden D Loan MV McCabe G McCabe L Daida YG Boushey CJ the ACT Research Team Hispanic and Asian pubertal girls have higher android/gynoid fat ratio than whites Obesity 2007 15 1565 1570 10.1038/oby.2007.185 17557994

[B16] Morrison JA Barton BA Obarzanek E Crawford PB Guo SS Schreiber GB Racial differences in the sums of skinfolds and percentage of body fat estimated from impedance in black and white girls, 9 to 19 years of age: The National Heart, Lung, and Blood Institute Growth and Health Study Obes Res 2001 9 297 305 10.1038/oby.2001.37 11346671

[B17] Malina RM Huang YC Brown KH Subcutaneous adipose tissue distribution in adolescent girls of 4 ethnic groups Int J Obes Relat Metab Disord 1995 19 793 797 8589780

[B18] Herd SL Gower BA Dashti N Goran M Body fat, fat distribution and serum lipids, lipoproteins and apolipoproteins in African-American and Caucasian-American prepubertal children Int J Obes 2001 25 198 204 10.1038/sj.ijo.0801524 11410820

[B19] Goran MI Nagy TR Treuth MS Trowbridge C Dezenberg C McGloin A Gower BA Visceral fat in white and African American prepubertal children Am J Clin Nutr 1997 65 1703 1708 9174463 10.1093/ajcn/65.6.1703

[B20] Tanaka S Horimai C Katsukawa F Ethnic difference in abdominal visceral fat accumulation between Japanese, African-Americans, and Caucasians: A meta-analysis Acta Diabetol 2003 40 Suppl 1 302 304 10.1007/s00592-003-0093-z 14618500

[B21] He Q Horlick M Thornton J Wang J Pierson RN Jr Heshka S Gallagher D Sex-specific fat distribution is not linear across pubertal groups in a multiethnic study Obes Res 2004 12 725 733 10.1038/oby.2004.85 15090643

[B22] Lear SA Humphries KH Kohli S Chockalingam A Frohlich JJ Birmingham CL Visceral adipose tissue accumulation differs according to ethnic background: results of the Multicultural Community Health Assessment Trial (M-CHAT) Am J Clin Nutr 2007 86 353 359 17684205 10.1093/ajcn/86.2.353

[B23] Tanner J Whitehouse R Clinical longitudinal standards for height, weight, height velocity, weight velocity, and stages of puberty Arch Dis Child 1976 51 170 179 10.1136/adc.51.3.170 952550 PMC1545912

[B24] Marfell-Jones M Olds T Stewart A Carter JEL International standards for anthropometric assessment (revised 2006) The International Society for the Advancement of Kinanthropometry 2006 2

[B25] Lohman TG Applicability of body composition techniques and constants for children and youths Exerc Sport Sci Rev 1986 14 325 357 3525188

[B26] Park YW Allison DB Heymsfield SB Gallagher D Larger amounts of visceral adipose tissue in Asian Americans Obes Res 2001 9 381 387 10.1038/oby.2001.49 11445659

[B27] Chandalia M Abate N Garg A Stray-Gundersen J Grundy SM Relationship between generalized and upper body obesity to insulin resistance in Asian Indian men J Clin Endocrinol Metab 1999 84 2329 2335 10.1210/jc.84.7.2329 10404798

[B28] Raji A Seely EW Arky RA Simmons DC Body fat distribution and insulin resistance in healthy Asian Indians and Caucasians J Clin Endocrinol Metab 2001 86 5366 5371 10.1210/jc.86.11.5366 11701707

[B29] Wu CH Heshka S Wang J Pierson RN Jr Heymsfield SB Laferrère B Wang Z Albu JB Pi-Sunyer X Gallagher D Truncal fat in relation to total body fat: influences of age, sex and ethnicity and fatness Int J Obes 2007 31 1384 1391 10.1038/sj.ijo.0803624 PMC2752389 17452992

[B30] Novotny R Daida YG Grove JS Achsrya S Vogt TM Formula feeding in infancy is associated with adolescent body fat and earlier menarche Cell Mol Biol 2003 49 1289 1293 14984000

[B31] Hughes K Aw T Kuperan P Choo M Central obesity, insulin resistance, syndrome X, lipoprotein (a), and cardiovascular risk in Indians, Malays, and Chinese in Singapore J Epidemiol Community Health 1997 51 394 399 10.1136/jech.51.4.394 9328546 PMC1060508

[B32] Daniels SR Khoury PR Morrison JA Utility of different measures of body fat distribution in children and adolescents Am J Epidemiol 2000 152 1179 1184 10.1093/aje/152.12.1179 11130624

[B33] Pouliot MC Despres JP Lemieux S Moorjani S Bouchard C Tremblay A Nadeau A Lupien PJ Waist circumference and abdominal sagittal diamerer: best simple anthropometric indexes of abdominal visceral adipose tissue accumulation and related cardiovascular risk in men and women Am J Cardiol 1994 73 460 468 10.1016/0002-9149(94)90676-9 8141087

[B34] Lee S Bacha F Gungor N Arslanian SA Waist circumference is an independent predictor of insulin resistance in black and white youths J Pediatr 2006 148 188 194 10.1016/j.jpeds.2005.10.001 16492427

[B35] Ng VW Kong AP Choi KC Ozaki R Wong GW So WY Tong PC Sung RY Xu LY Chan MH Ho CS Lam CW Chan JC BMI and waist circumference in predicting cardiovascular risk factor clustering in Chinese adolescents Obesity 2007 15 494 503 10.1038/oby.2007.588 17299123

[B36] Lofren I Herron K Zern T West K Patalay M Shachter NS Waist circumference is a better predictor than body mass index for coronary heart disease risk in overweight premenopausal women J Nutr 2004 134 1071 1076 15113947 10.1093/jn/134.5.1071

[B37] Janssen I Katzmarzyk P Ross R Waist circumference and not body mass index explains obesity-related health risk Am J Clin Nutr 2004 79 379 384 14985210 10.1093/ajcn/79.3.379

[B38] Alberti K Zimmet P Shaw J Metabolic syndrome - a new world-wide definition. A consensus statement from the international diabetes federation Diabet Med 2006 23 469 480 10.1111/j.1464-5491.2006.01858.x 16681555

[B39] Zimmet P Alberti G Kaufman F Tajima N Silink M Arslanian S Wong G Bennett P Shaw J Caprio S The metabolic syndrome in children and adolescents Lancet 2007 369 2059 2061 10.1016/S0140-6736(07)60958-1 17586288

[B40] Liu A Hills AP Hu X Li Y Du L Xu Y Byrne NM Ma G Waist circumference cut-off points for the prediction of cardiovascular risk factors clustering in Chinese school-aged children: a cross-sectional study BMC Public Health 2010 10 82 10.1186/1471-2458-10-82 20170510 PMC2836988

[B41] Sung RY So H Choi K Nelson EA Li AM Yin JA Kwok CW Ng P Fok T Waist circumference and waist-to-height ratio of Hong Kong Chinese children BMC Public Health 2008 8 324 333 10.1186/1471-2458-8-324 18808684 PMC2563004

[B42] Ji C Sung R Ma G Ma J He Z Chen T Waist circumference distribution of Chinese school-age children and adolescents Biomed Environ Sci 2010 23 12 20 10.1016/S0895-3988(10)60026-8 20486431

[B43] Poh BK Jannah AN Chong LK Ruzita AT Ismail MN Mccarthy D Waist circumference percentile curves for Malaysian children and adolescents aged 6.0 - 16.9 years Int J Pediatr Obes 2011 in press 10.3109/17477166.2011.583658 21668385

[B44] Kimm S Barton B Obarzanek E McMahon R Sabry Z Waclawiw M Schreiber G Morrison J Similo S Daniels S Racial divergence in adiposity during adolescence: The NHLBI growth and health study Pediatrics 2001 107 30 36 10.1542/peds.107.1.30 11230615

[B45] Wang J Thornton JC Bari S Williamson B Gallagher D Heymsfield SB Horlick M Kotler D Laferrère B Mayer L Pi-Sunyer FX Pierson RN Jr Comparisons of waist circumferences measured at 4 sites Am J Clin Nutr 2003 77 379 384 12540397 10.1093/ajcn/77.2.379

[B46] Orphanidou C McCargar L Birmingham CL Mathieson J Goldner E Accuracy of subcutaneous fat measurement: comparison of skinfold calipers, ultrasound, and computed tomography J Am Diet Assoc 1994 94 855 858 10.1016/0002-8223(94)92363-9 8046177

[B47] Hayes PA Sowood PJ Belyavin A Cohen J Simth FW Subcutaneous fat thickness measured by magnetic resonance imaging, ultrasound, and calipers Med Sci Sports Exerc 1988 20 303 309 10.1249/00005768-198806000-00015 3386511

